# An intracellular phosphorus-starvation signal activates the PhoB/PhoR two-component system in *Salmonella enterica*

**DOI:** 10.1128/mbio.01642-24

**Published:** 2024-08-06

**Authors:** Roberto E. Bruna, Christopher G. Kendra, Mauricio H. Pontes

**Affiliations:** 1 Department of Pathology and Laboratory Medicine, Pennsylvania State University College of Medicine, Hershey, Pennsylvania, USA; 2 The One Health Microbiome Center, Huck Institute of the Life Sciences, Pennsylvania State University, Camp Hill, Pennsylvania, USA; Instituto Gulbenkian de Ciência, Oeiras, Portugal; Ludwig-Maximilians-Universitat Munchen Bereich Mikrobiologie, Martinsried/Planegg, Germany

**Keywords:** PhoB, PhoR, phosphate, phosphorus, *Salmonella enterica*, cytoplasmic signal, signal transduction

## Abstract

Bacteria acquire P primarily as inorganic orthophosphate (Pi, PO_4_^3−^). Once internalized, Pi is rapidly assimilated into biomass during the synthesis of ATP. Because Pi is essential, but excessive ATP is toxic, the acquisition of environmental Pi is tightly regulated. In the bacterium *Salmonella enterica* (*Salmonella*), growth in Pi-limiting environments activates the membrane sensor histidine kinase PhoR, leading to the phosphorylation of its cognate transcriptional regulator PhoB and subsequent transcription of genes involved in adaptations to low Pi. Pi limitation promotes PhoR kinase activity by altering the conformation of a membrane signaling complex comprised of PhoR, the multicomponent Pi transporter system PstSACB and the regulatory protein PhoU. However, the identity of the Pi-starvation signal and how it controls PhoR activity remain unknown. Here, we identify conditions where the PhoB and PhoR signal transduction proteins can be maintained in an inactive state when *Salmonella* is grown in media lacking Pi. Our results demonstrate that PhoB/PhoR is activated by an intracellular P-insufficiency signal.

**IMPORTANCE:**

In enteric bacteria, the transcriptional response to phosphorus (P) starvation is controlled by a specialized signal transduction system comprised of a membrane-bound, multicomponent signal sensor, and a cytoplasmic transcriptional factor. Whereas this system has been primarily studied in the context of phosphate (Pi) starvation, it is currently unknown how this stress initiates signal transduction. In the current study, we establish that this signaling system is regulated by a cytoplasmic signal arising from insufficient P. We demonstrate that rather than responding to extracellular conditions, cells couple the activation of their P starvation response to the availability of cytoplasmic P. This regulatory logic may enable cells to prevent toxicity resulting from excessive Pi acquisition and hinder the onset of a P starvation response when their metabolic demands are being met through the consumption of P sources other than Pi.

## INTRODUCTION

Phosphorous (P) is essential for life. This element is an integral component of lipids, nucleotides, and nucleic acids, and can account for as much as 5.5% of the dry weight of actively growing cells (1, 2). In nature, the vast majority of P exists as inorganic orthophosphate (Pi, PO_4_^3−^), either as hydrated phosphate minerals or soluble phosphate ions. Pi is the preferred P source of most living cells, being normally assimilated into biomass during the synthesis of adenosine triphosphate (ATP). ATP functions as the main Pi donor molecule and the primary source of high-energy phosphoanhydride bonds that are used to power energy-dependent processes. Cells regulate Pi uptake to obtain adequate supplies of this nutrient and avoid toxicity resulting from accumulation of intracellular Pi and uncontrollable ATP synthesis (3–6).

In *Escherichia coli*, *Salmonella enterica* (*Salmonella*), and closely related bacterial species, the response to P starvation is governed by the PhoB/PhoR two-component signal transduction system. Growth in environments containing limiting Pi promotes auto-phosphorylation of the membrane-bound histidine kinase/phosphatase PhoR, and subsequent transfer of the phosphoryl group to the cytoplasmic response regulator PhoB. The phosphorylation of PhoB favors an active dimerization state in which this transcription factor can efficiently bind to DNA and modulate the transcription of target genes (7–11). Active PhoB promotes the transcription of genes encoding proteins that scavenge P from the environment and cellular components (e.g., *phoE*, *pstSCAB*, *ugpBAECQ*, and *waaH*) and alleviate secondary stresses generated by P insufficiency (e.g., *katE* and *cydB*) such as oxidative, envelope, and acetic acid stresses (12–21).

**Fig 1:**
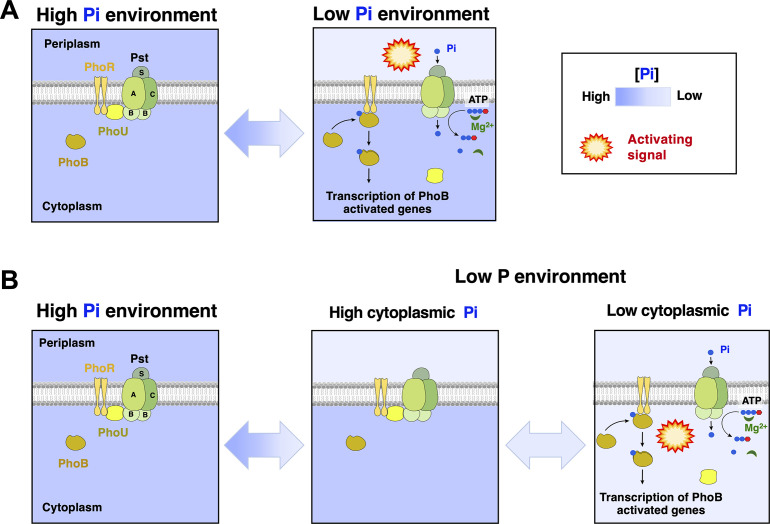
Environmental control of PhoB/PhoR. Schematics depicting environmental activation of PhoB/PhoR by extracellular Pi (**A**) or an intracellular signal resulting from P-insufficiency (**B**). (**A**) Growth in Pi-abundant conditions maintains the PhoR-PhoU-P PstSACB signaling complex in an inactive state. A decrease in extracytoplasmic Pi concentration changes the conformation of the signaling complex, leading to PhoB/PhoR activation and transcription of PhoB-dependent genes, such as the *pstSCAB-phoU* operon. (**B**) Growth in Pi-abundant conditions maintains the PhoR-PhoU-PstSACB signaling complex in an inactive state. When extracytoplasmic Pi concentrations decrease, cells may be able to maintain their intracellular Pi levels through housekeeping Pi transporters such as PitA. When Housekeeping Pi transporters are unable to maintain intracellular Pi levels, cells experience a decrease in the concentration of intracellular Pi, leading to the activation of PhoB/PhoR.

Prototypical sensor histidine kinases often harbor an extracytoplasmic domain that participates in the recognition of a cognate regulatory molecule or signal that regulates signal transduction (22–24). PhoR is unusual because (i) it lacks a sizable extracytoplasmic domain and (ii) its activity is controlled by physical interactions with the high-affinity Pi transporter PstSCAB that are mediated by the cytoplasmic protein PhoU (25–28) (Fig. 1). Growth in P-limiting conditions is thought to alter these physical interactions, fostering the kinase state of PhoR, phosphorylation of PhoB and the subsequent activation of the PhoB regulon (28–30). However, how this regulatory cascade begins is currently unknown. PhoR could be repressed by extracytoplasmic Pi acting on the Pst components of the signaling complex (Fig. 1A), or by a P-sufficiency signal affecting the cytoplasmic portion of the complex where PhoR interacts with PhoU through a Per-Arnt-Sim (PAS) domain (Fig. 1B) (3, 4, 27–37).

We previously established that *E. coli* and *Salmonella* PhoB/PhoR systems can be activated in the presence of high Pi, when these organisms undergo translational arrest resulting from either cytoplasmic Mg^2+^ starvation, or treatment with antibiotics that inhibit protein synthesis (3, 35, 36). These results implied that this two-component system responds to an intracellular signal that is normally generated during growth in low Pi but that can be brought about by these other physiological disturbances. In the current study, we conduct experiments to specifically test this hypothesis. We show that if *Salmonella* is provided with an alternative P source that is metabolized in a PhoB-independent manner, PhoB/PhoR remains inactive during growth in Pi-free media, thereby demonstrating that PhoB/PhoR activity is repressed by an intracellular P sufficiency signal (Fig. 1B).

## RESULTS

### Pi transporters influence PhoB/PhoR activity during growth in high Pi environments

When *Salmonella* is subjected to a nutritional downshift from medium containing high Pi to medium lacking a P source, the concentration of free intracellular Pi decreases (33), PhoB/PhoR is rapidly activated and genes promoting P homeostasis are transcribed (15). PhoB/PhoR is also activated when *Salmonella* is incubated in high Pi medium while being subjected to translation arrest, a cellular state where ribosomes can no longer recycle Pi from ATP and GTP during translation elongation (36). Whereas this suggests that PhoB/PhoR responds to an intracellular signal that fluctuates with the concentration of intracellular Pi, we sought to specifically test this hypothesis by measuring the effects of disrupting intracellular Pi levels on PhoB/PhoR activity.

**Fig 2:**
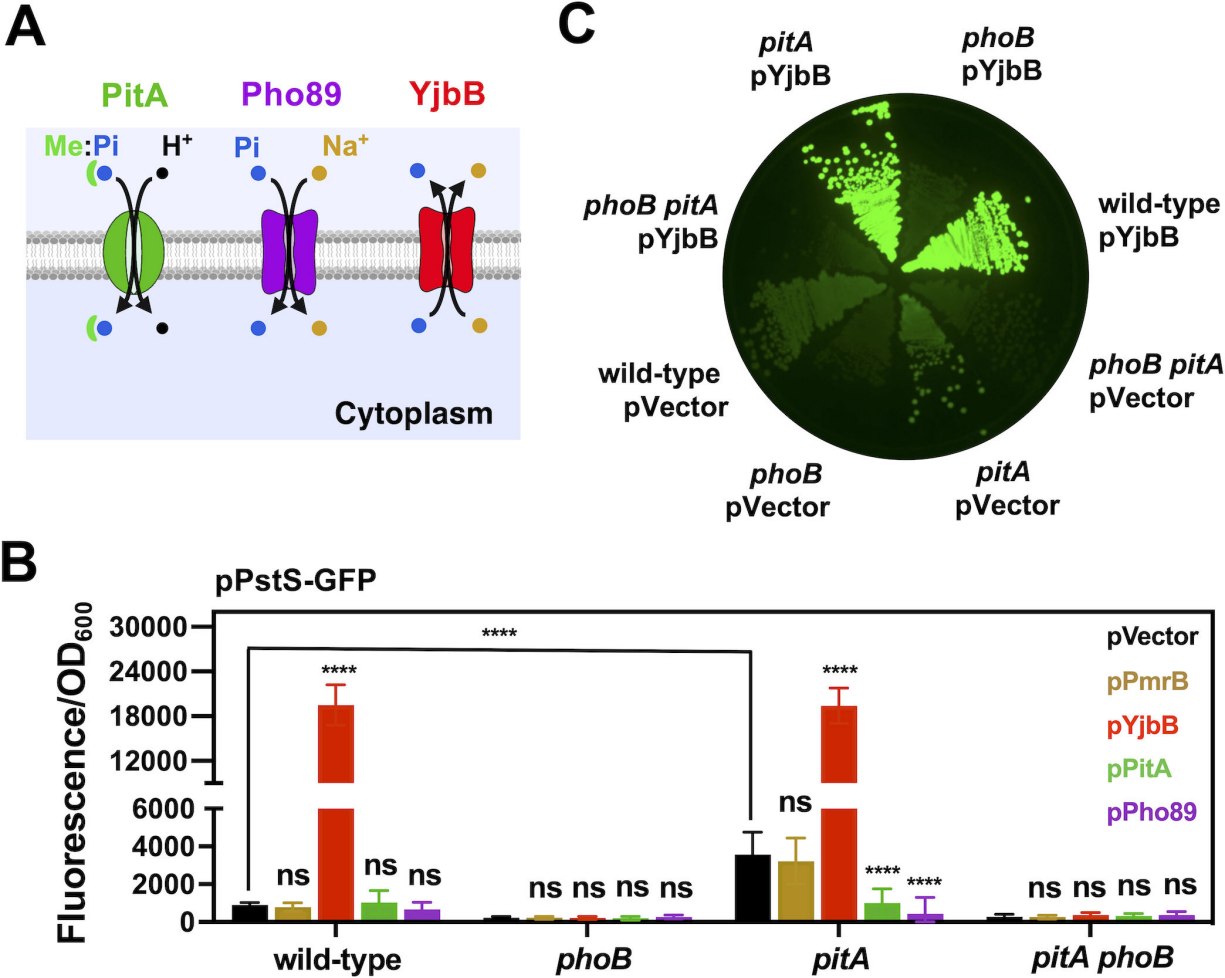
Expression of Pi importers and exporters modulates PhoB/PhoR signaling in opposite manners. (**A**) Cartoon representations of Pi transporter systems used in the described experiments. PitA uses the proton motive force to transport soluble, neutral divalent metal:phosphate complexes (Me:Pi) into the cytoplasm. Pho89 is a high-affinity Na^+^/Pi co-transporter from the yeast *Saccharomyces cerevisiae*. YjbB is a Na^+^/Pi co-transporter that has been shown to promote Pi export. (**B**) Fluorescence derived from wild-type (14028s), *phoB* (EG9054), *pitA* (MP1251), and *pitA phoB* (MP2405) *Salmonella* carrying pPstS*-*GFP (pACYC-P*pstS*-GFPc) and either pVector (pUHE-21), pPmrB (pUHE-PmrB), pYjbB (pUHE-YjbB), pPitA (pUHE-PitA), or pPho89 (pUHE-Pho89). Experiments were conducted in LB broth. The expression of YjbB, PitA, and Pho89 were induced after 2 h of growth by the addition of 250 µM of isopropyl β-d-1-thiogalactopyranoside (IPTG). Normalized GFP values (fluorescence/OD_600_) were determined after 24 h of growth. Means ± SDs of four independent experiments are shown. ****P < 0.0001; ns, no significant difference. Two-way ANOVA calculated with Dunnett multiple-comparison test. Unless indicated otherwise, statistical comparisons were made against control (pVector) samples for each of the genotypes assayed. (**C**) Fluorescence derived from wild-type (14028s), *pitA* (MP1251), and *phoB* (EG9054) *Salmonella* carrying plasmids pPstS*-*GFP (pACYC-P*pstS*-GFPc) and either pVector (pUHE-21) or pYjbB (pUHE-YjbB). Cells were grown on LB-agar plates containing 100 µM of IPTG to induce YjbB expression. Plates were incubated at 37°C during 16–18 h. Images are representative of three independent experiments.

First, we measured the effect of the housekeeping Pi transporter PitA on PhoB/PhoR activity (Fig. 2A). We determined that *pitA* deletion resulted in a small, but significant and reproducible rise in the fluorescence derived from a PhoB-activated P*pstS-gfp* transcription reporter fusion. This occurred even during *Salmonella* growth in Pi-rich media (12), where PhoB/PhoR is normally inactive (Fig. 2B; Fig. S1) (15). The elevated P*pstS-gfp* activity in the *pitA* deletion strain was repressed by ectopically expressing the *pitA* gene from a plasmid, but not by the empty plasmid control (Fig. 2B). The increased *pstS* transcription conferred by *pitA* mutation resulted from the activation of PhoB by decreased intracellular Pi levels. This is because P*pstS-gfp*-derived fluorescence was abolished by either a *phoB* deletion or ectopic plasmid expression of *pho89*, encoding a Pit-like phosphate transporter from the budding yeast *Saccharomyces cerevisiae* (38–41) (Fig. 2A and B). In contrast, P*pstS-gfp* activity was not impacted by ectopic plasmid expression of *pmrB*, encoding an inner-membrane protein that does not participate in Pi metabolism (42) (Fig. 2B) Control measurements indicated protein expression from these plasmids did not perturb bacterial growth rate (Fig. S2).

Second, we sought to measure the effect of exporting cytoplasmic Pi on PhoB/PhoR activity. We determined that, during growth in Pi-rich medium, ectopic plasmid expression of the Pi exporter *yjbB* (43) (Fig. 2A) resulted in 20- and 5-fold increases in P*pstS-gfp* activity in wild-type and *pitA* cells, respectively, in comparison with isogenic strains harboring the empty vector or the PmrB-expressing control plasmid (Fig. 2B and C). This phenomenon was independent of bacterial growth rate (Fig. S2) and was abolished by the introduction a *phoB* deletion (Fig. 2B and C). Taken together, these results indicate that PhoB/PhoR can be repressed by proteins that import Pi, and activated by proteins that export Pi out of the cytoplasm during growth in Pi-abundant conditions.

### Utilization of alternative P sources represses PhoB/PhoR activity in the absence of exogenous Pi

**Fig 3:**
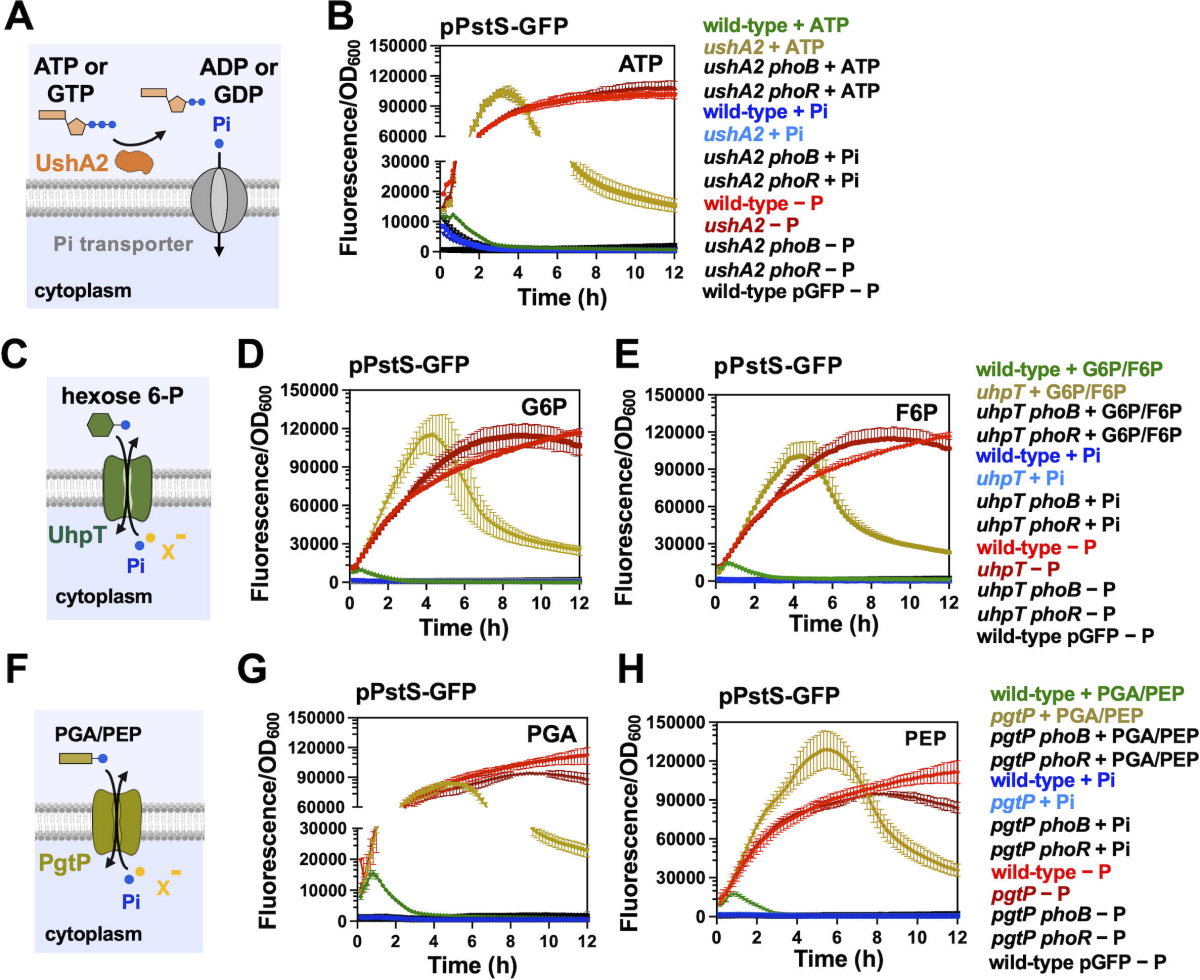
Effect of organic-P utilization on PhoB/PhoR activity. (**A**) Schematics of *Salmonella* UshA2 protein. UshA2 is a periplasmic 5′ nucleotidase capable of extracting Pi from nucleotides such as ATP, ADP, AMP, and GTP (15). (**B**) Fluorescence from wild-type (14028s), *ushA2* (MP1779), *ushA2 phoB* (MP2260), and *ushA2 phoR* (MP2261) strains of *Salmonella* carrying pPstS*-*GFP (pACYC-PstS-GFPc) or a promoterless GFP vector control pGFP ( pACYC-GFPc) in MOPS medium containing either no P source (−P), 1 mM of K_2_HPO_4_ (Pi) or 1 mM of ATP. The activity of P*pstS-gfp* fusion is different in wild-type and *ushA2* when these strains are grown with ATP as the sole P source (*P* < 0.001; modified chi-squared method) (44). (**C**) Schematic representation of *Salmonella* UhpT protein. UhpT exchanges extracellular hexose-P compounds, such as glucose 6P (G6-P) and fructose 6P (F6-P) with intracellular Pi or similar anions (X^−^), thereby supporting the growth of *Salmonella* on G6-P or F6-P as sole P sources (15). (**D and E**) Fluorescence from wild-type (14028s), *uhpT* (MP1738), *uhpT phoB* (MP2264), and *uhpT phoR* (MP2265) strains of *Salmonella* carrying pPstS*-*GFP or pGFP in MOPS medium containing either no P source (−P), 1 mM of K_2_HPO_4_ (Pi) or 1 mM of either G6-P (**D**) or F6-P (**E**). P*pstS-gfp* activity is different in wild-type and *uhpT Salmonella* strains during growth on either G6P or F6P as the sole P source (*P* < 0.001; modified chi-squared method) (44). (**F**) Schematic representation of *Salmonella* PgtP protein. PgtP exchanges extracellular phosphoenolpyruvate (PEP) or 2- or 3-phosphoglycerate (PGA) with intracellular Pi or similar anions (X^−^) and promotes *Salmonella* growth on these substrates as the sole P sources (15). (**G and H**) Fluorescence from wild-type (14028s), *pgtP* (MP1739), *pgtP phoB* (MP2262), and *pgtP phoR* (MP2263) strains of *Salmonella* carrying pPstS*-*GFP or pGFP in MOPS medium containing either no P source (−P), 1 mM of K_2_HPO_4_ (Pi) or 1 mM of either PGA (**G**) or PEP (**H**). P*pstS-gfp* emitted fluorescence is different in wild-type and *pgtP* strains during growth with either PGA or PEP as the sole P source (*P* < 0.001; modified chi-squared method) (44). Prior to fluorescence readings, cultures were grown to mid-logarithmic phase (OD_600_ ≈ 0.4) in MOPS medium containing 1 mM K_2_HPO_4_, washed in MOPS medium lacking a P source and resuspended in MOPS medium containing either no P source (−P) or containing the indicated P source. In all cases, means ± SDs of at least three independent experiments are shown.

We have recently established that *Salmonella* can utilize a variety of organic-P substrates as sole P source. This bacterium uses the UhpT transporter to import hexose-6-phosphate such as glucose-6-phosphate (G6-P) or fructose-6-phosphate (F6-P); the PgtP transporter to import 3-phosphoglyceric acid (PGA) and phosphoenolpyruvate (PEP), and the periplasmic 5′-nucleotidase UshA2 to release terminal Pi from nucleotides such as ATP or GTP (Fig. 3) (15). Because the expression and activity of these proteins is independent of PhoB (15), we sought to test if growth on these organic-P substrates could inhibit PhoB/PhoR activity. Specifically, we reasoned that if PhoB/PhoR senses an intracellular signal that is generated during P starvation, then P*pstS-gfp* activity should remain low in wild-type cells that are shifted from Pi-rich medium to media that lacks Pi but contain an alternative organic-P source that is catabolized in a PhoB-independent fashion (e.g., G6-P, F6-P, PGA, PEP, or ATP) (Fig. 3A, C and F). However, P*pstS-gfp* fluorescence should increase in mutants that are unable to efficiently utilize these substrates, given that they are expected to experience a decrease in cytoplasmic Pi (Fig. 1) (33).

Accordingly, we subjected exponentially growing wild-type, *uhpT*, *pgtP*, and *ushA2 Salmonella* strains to a shift from Pi-rich medium to media containing either G6-P, F6-P, PGA, PEP, or ATP as the sole P source. Despite the lack of exogenous Pi in the growth media, P*pstS-gfp* activity remained low in wild-type cells (Fig. 3B, D, E, G and H). In contrast, P*pstS-gfp* fluorescence increased when the *uhpT*, *pgtP*, and *ushA2* mutant strains were shifted to media containing G6-P/F6-P, PGA/PEP, or ATP, respectively (Fig. 3B, D, E, G and H). Regardless of the genetic background, P*pstS-gfp* activity remained low following shifts to fresh Pi-rich medium and increased following shifts to P-deficient medium (Fig. 3B, D, E, G and H). P*pstS-gfp* fluorescence was abolished by mutations in either *phoB* or *phoR*, indicating that the observed changes resulted from PhoB/PhoR activity (Fig. 3B, D, E, G and H). Importantly, whereas these nutritional shifts yield disparities in growth rates across genetic backgrounds (Fig. S3A through C), we determined that P*pstS-gfp* activity was independent of the growth rate (Fig. S3D and E). Taken together, these results are consistent with the notion that PhoB/PhoR is activated when cytoplasmic levels of P are insufficient.

### Transcriptional rewiring of PhoB-dependent *ugpBAECQ* allows PhoB/PhoR repression by Gly-3P metabolism

While UshA2 releases Pi from nucleotides at the periplasm, the transporters UhpT and PgtP can mediate the exchange of cytoplasmic Pi with their cognate organic-P substrates (45–48). Hence, during growth on the aforementioned organic-P substrates, UshA2, UhpT, and PgtP could repress PhoB/PhoR through endogenously generated Pi that is either released at (UshA2) or exported to (UhpT and PgtP) the periplamic space. To further investigate this possibility, we decided to take advantage of the two well-characterized *sn*-glycerol-3-P (Gly-3P) transport systems that are present in *Salmonella: ugpBAECQ* and *glpT*.

**Fig 4:**
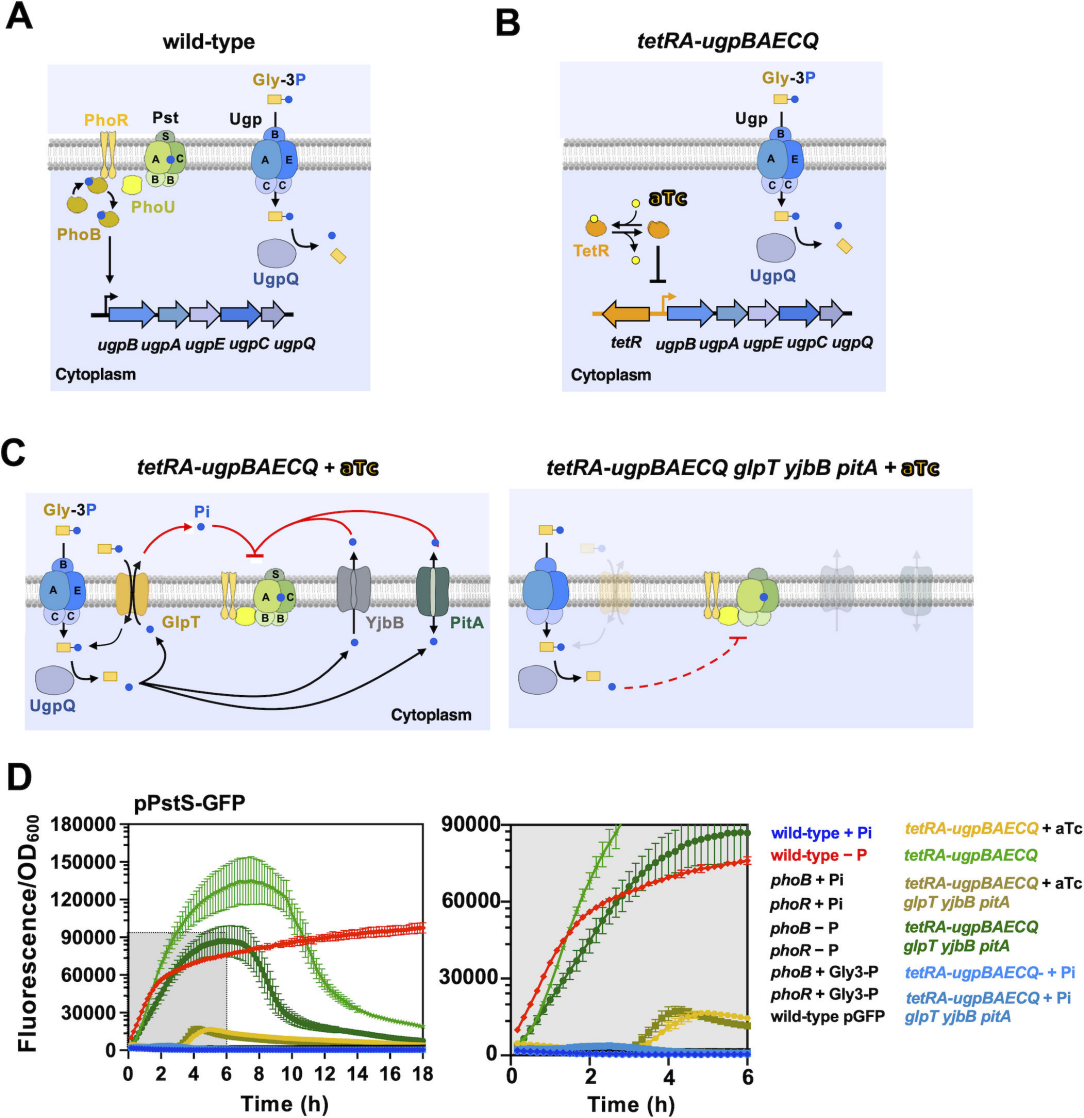
Effect of Gly-3P metabolism on PhoR activity. (**A**) Cartoon representation depicting the native regulatory circuit whereby PhoB/PhoR promotes the transcription of the *ugpBAECQ* operon. Genes encoded in this operon mediate the uptake and degradation of *sn*-glycerol 3-phosphate (Gly-3P). (**B**) Cartoon representation depicting the engineered regulatory circuit whereby TetR controls the transcription of the *ugpBAECQ* operon in response to anhydrotetracycline (aTc). The growth of the resulting *tetRA-ugpBAECQ Salmonella* strain (MP2133) on Gly-3P depends on the presence of aTc (Fig. S4). (**C**) Cartoon representations depicting the pathways for the movement of Pi across the cytoplasmic membrane in the *tetRA-ugpBAECQ* (MP2133; left-hand-side panel) and *tetRA-ugpBAECQ glpT yjbB pitA* (MP2285; right-hand-side panel) strain grown on Gly-3P in the presence of aTc. (**D**) Fluorescence from *tetRA-ugpBAECQ* (MP2133) and *tetRA-ugpBAECQ glpT yjbB pitA* (MP2285) carrying pPstS*-*GFP (pACYC-PstS-GFPc) or a promoterless GFP vector control pGFP (pACYC-GFPc ). Strains were grown overnight in MOPS medium containing 2 mM K_2_HPO_4_ and the presence or absence of 0.5 µg/mL of aTc. Prior to fluorescence readings, cells were washed three times with MOPS-glucose lacking a P source and inoculated (1:100) into fresh MOPS medium containing either 1 mM K_2_HPO_4_ (+Pi), or 1 mM of Gly-3P in the presence or absence of 0.5 µg/mL of aTc. Fluorescence of wild-type (14028s), *phoB* (EG9054), and *phoR* (MP2256) strains harboring pPstS*-*GFP and grown in the presence of 1 mM K_2_HPO_4_ or absence of P (−P) is shown, along with that of wild-type (14028s) harboring pGFP during growth in MOPS-glucose medium lacking P. Rightmost panel shows enlarged portion of the figure shaded in gray. Note that P*pstS-gfp* emitted fluorescence pattern is significantly different between *tetRA-ugpBAECQ* (MP2133) cultures grown in the presence and absence of aTc, and between *tetRA-ugpBAECQ* (MP2133) and *tetRA-ugpBAECQ glpT yjbB pitA* (MP2285) when they are grown in medium lacking aTc (in both instances, *P* < 0.001; modified chi-squared method) (44). The graph depicts representative fluorescence measurements derived from at least three experiments.

The *ugpBAECQ* locus encodes for an ATP-dependent Gly-3P importer (UgpBAEC) and a cytoplasmic phosphodiesterase that is capable of extracting Pi from Gly-3P (UgpQ). This operon is transcriptionally activated by PhoB and it is required for growth on Gly-3P as sole P source (15) (Fig. 4A). In comparison, *glpT* encodes a PhoB-independent Gly-3P/Pi antiporter that balances the intracellular concentrations of Pi through exchange reactions with either intra- or extracytoplasmic Gly-3P molecules. When Gly-3P is the sole P source, Gly-3P is taken up and metabolized via the proteins encoded by the *ugpBAECQ* locus. Once cytoplasmic Pi accumulates, GlpT mediates the exchange between cytoplasmic Pi and extracytoplasmic Gly-3P (33).

To uncouple Gly-3P utilization from PhoB/PhoR activity, we constructed a *Salmonella* strain in which the PhoB-activated promoter upstream of the *ugpBAECQ* operon was replaced by a *tetA* promoter and a divergently transcribed *tetA* repressor encoded by the *tetR* gene (*tetRA-ugpBAECQ*). In this strain, *ugpBAECQ* is transcribed, and Gly-3P is metabolized, in response to anhydrotetracycline (aTc) (Fig. 4B; Fig. S4). Hence, if PhoB/PhoR responds to an intracellular signal, then P*pstS-gfp* activity should be damped when *tetRA-ugpBAECQ* harboring strains are grown in medium containing aTc and Gly-3P as the sole P source. However, PhoB/PhoR and P*pstS-gfp* activity should increase if aTc is removed. This behavior should be independent of Pi exporting pathways requiring GlpT, YjbB, and PitA (Fig. 4C).

Accordingly, we grew a *tetRA-ugpBAECQ* strain in medium containing high Pi, in the presence or absence of aTc, and subsequently shifted the cells to the same medium containing Gly-3P as the sole P source. In cultures grown in the absence of aTc, the fluorescence derived from the PhoB-dependent P*pstS-gfp* reporter began to increase immediately after shifting cells from Pi to Gly-3P, reaching approximately 100-fold above background levels after 12 h (Fig. 4D). In contrast, in cultures grown in the presence of aTc, P*pstS-gfp*-derived fluorescence remained at background levels for the initial 4 h, subsequently increasing to approximately 10-fold above background levels after 6 h of growth (Fig. 4D). Consistent with the hypothesis that PhoB/PhoR senses an intracellular P starvation signal, introduction of *glpT yjbB pitA* null mutations in the *tetRA-ugpBAECQ* strain decreased P*pstS-gfp* activity during growth in Gly-3P medium lacking aTc (Fig. 4D). As expected, P*pstS-gfp*-derived fluorescence remained at background levels in strains carrying either a *phoB* or a *phoR* null mutation (Fig. 4D). Taken together, these results demonstrate that the PhoB/PhoR signal transduction system is controlled by a cytoplasmic P starvation signal.

## DISCUSSION

In the current study, we show that PhoB/PhoR signal transduction is inhibited by the activity of PitA and Pho89, two proteins that import Pi into the cytoplasm; and is activated by overexpression of YjbB, a Pi exporter. We establish that the metabolism of several organic-P substrates represses PhoB/PhoR in media lacking exogenous Pi. Finally, we demonstrate that when the *ugpBAECQ* operon, encoding Gly3-P metabolizing proteins, is transcriptionally uncoupled from PhoB/PhoR activation, Gly3-P metabolism inhibits PhoB/PhoR activity. Inhibition by Gly3-P occurs even in *glpT pitA yjbB* triple mutant strains, which lack proteins that can mediate Pi export.

The PhoB/PhoR-mediated response to P starvation begins with the detection of a low P signal by the PhoR signaling complex at the membrane. In this complex, PhoR physically interacts with the PstB component of the PstSCAB Pi transporter via the PhoU protein (28). Deletion of *phoU*, *pstA*, *pstB*, *pstS*, or *pstSCAB-phoU* fosters the kinase state of PhoR and the transcription of PhoB-dependent genes even when cells are grown in Pi-rich conditions (28, 29, 34, 49). Whereas this indicates that protein interactions that are formed in Pi-rich conditions maintain PhoR in an inactive state, and that Pi levels may be sensed by components of the complex other than PhoR, it also suggests a way in which this complex may sense low Pi. That is, PhoR activity could be controlled by conformations adopted by the other components of the signaling complex in response to either the binding of extracytoplasmic Pi to PstSCAB transport components, or the movement of extracytoplasmic Pi through the transport system (26, 28–30, 32). However, because Pi starvation regimens that are often used to study this two-component system decrease the intracellular concentration of Pi (33), the above model cannot rule out the possibility that PhoR is activated by a signal sensed at the cytoplasmic face of the complex.

Noteworthily, a number of previous studies suggest that the PhoR signaling complex may respond to an intracellular signal. First, whereas inactivation of *pstS* promotes the kinase state of PhoR during growth of *E. coli* in Pi-rich medium, ectopic overexpression of phosphate transporters PitA or PitB represses PhoR kinase activity. This implies that an increase in cytoplasmic Pi can compensate for the defect conferred by the *pstS* deletion on the signaling complex (34). Second, in certain purine auxotrophic strains of *E. coli*, conditions which cause an expansion of adenine nucleotides activate PhoR during growth in otherwise Pi-rich medium (31). This indicates that PhoR activation can result from physiological disturbances taking place in the cytoplasm. Third, treatment of *E. coli* or *Salmonella* with translation inhibitors causes the expansion of ATP pools and promotes PhoR activation in high Pi media (36). Here, activation seems to result from a reduction in pools of free cytoplasmic Pi that are drained due to ATP accumulation, rather than to ATP itself. This is because PhoR remains inactive when translation-arrested cells hydrolyze excess ATP (36) or accumulate Pi alongside with ATP (3). This notion is further supported by the physiological response of cells to P insufficiency: P starvation leads to the accumulation of (p)ppGpp (50, 51) which, in turn, inhibits nucleotide biosynthesis (15, 52). That is, P starvation induces PhoB/PhoR activation and a concomitant reduction in the levels of ATP, GTP, and other nucleotides.

It has been claimed that PhoB/PhoR is activated in response to a decrease in extracellular Pi concentration (29). Whereas this would suggest that PhoB/PhoR behaves like the KdpD/KdpE or the PhoP/PhoQ two-component signal transduction systems by sensing extra- and intracellular signals (53–60), we are unable to find published experimental evidence supporting this claim (29). In contrast, our current study provides two sets of genetic evidence indicating that PhoB/PhoR signal transduction originates from a cytoplasmic signal. First, we demonstrate that PhoB/PhoR activity can be influenced by the expression of proteins that alter the cytoplasmic concentration of Pi. Whereas deletion of the gene encoding the housekeeping PitA transporter increases PhoB/PhoR activity during growth in high Pi medium, ectopic PitA expression represses PhoB/PhoR activity during growth in low Pi medium. This latter phenotype is also observed during ectopic expression of Pho89, a Pi transporter from *S. cerevisiae*, indicating that PhoR repression results from an increase in the levels of cytoplasmic Pi and not by another property of the native PitA transporter. In contrast to previous studies (34), our experiments show that Pi transporters, such as PitA, repress PhoB/PhoR activity in cells harboring an intact PhoR-PhoU-PstSACB signaling complex. Complementarily, during growth in high Pi medium, PhoB/PhoR can be activated by ectopic expression of the Pi exporter YjbB.

Second, the protein products encoded by *uhpT*, *pgtP*, and *ushA2* allow *Salmonella* to efficiently utilize Pi groups from organic substrates in a manner that is independent of both PhoB and Pi availability (15). Growth on these substrates as the sole P source supplies Pi to the cytoplasm and maintains PhoB/PhoR in an inactive state. Similarly, the proteins encoded by the PhoB-activated *ugpBAECQ* operon allow the importation and extraction of Pi from cytoplasmic Gly-3P (15, 33). When this operon is placed under the control of an aTc-inducible promoter, the addition of aTc quenches PhoB/PhoR activity in medium containing Gly-3P as the sole P source. Interestingly, when cells are grown in the absence of aTc, deletions of *glpT*, *pitA*, and *yjbB*, encoding proteins that can export cytoplasmic Pi under these growth conditions, lowers PhoB/PhoR activation. This implies that increased retention of cytoplasmic Pi in this strain dampens PhoB/PhoR activity.

Taken together, the results presented in this study establish that PhoB/PhoR activity is controlled by the concentration of cytoplasmic P, which exists primarily as Pi. In this context, the sensing of an intracellular signal may explain why this signal transduction system is activated when *Salmonella* is grown in medium containing excessive Pi while experiencing either cytoplasmic Mg^2+^ starvation or sodium chloride-induced hyperosmotic stress (61) (Fig. S5). Because both stresses hinder translation, thereby preventing the recycling of Pi from nucleotide triphosphates such as ATP and GTP, they are expected to lower intracellular Pi levels (3, 35, 36, 62). Furthermore, given that excessive Pi is toxic, activation of PhoB/PhoR by a cytoplasmic Pi starvation signal may serve as an effective safeguard against toxicity arising from PhoB/PhoR hyperactivation (3–5, 26, 27, 35, 36). That is, control of PhoB/PhoR by a cytoplasmic signal enables cells to maintain balanced growth by meeting their biosynthetic demands without exceeding their requirements for Pi. This general regulatory strategy has been adopted by other living organisms such as distantly related Gram-positive bacterial species and eukaryotic microbes such as *S. cerevisiae* (63–66).

## MATERIALS AND METHODS

### Bacterial strains, plasmids, oligonucleotides, and strains construction

The bacterial strains and plasmids used in this study are listed in Table S1 and oligonucleotide sequences are presented in Table S2. All *S. enterica* serovar Typhimurium strains are derived from 14028s pedigree (67) and were constructed by lambda Red-mediated recombineering (68). Deletions and gene fusions generated via this method were subsequently moved into clean genetic backgrounds via phage P22-mediated transduction as described (69). The Δ*phoR*::Km^R^ allele was derived from P22-lysate obtained from the defined gene deletion mutant library of *S. enterica* (70) (clone E02 from BEI Plate NR-29408). Bacterial strains used in recombination and transduction experiments were grown in Luria–Bertani (LB) medium at 30°C or 37°C (68, 69). When required, the LB medium was supplemented with ampicillin (100 µg/mL), chloramphenicol (20 µg/mL), kanamycin (50 µg/mL), gentamicin (18 µg/mL), apramycin (80 µg/mL) or tetracycline (20 µg/mL).

### Construction of *tetRA-ugpBAECQ* insertion

Phusion High-Fidelity DNA Polymerase (New England Biolabs) and primers 2075 and 2076 were used to PCR-amplify the *km^R^-tetRA* fragment from plasmid pBbB2K-GFP (71). The PCR product was inserted into the chromosome of *S. enterica* using lambda Red-mediated recombineering (68). The location of the insertion in kanamycin-resistant clones was verified by PCR using primer pairs 2077/2078 and 2079/2080. These clones were also tested for the ability to grow on Gly-3P as the sole P source, in the presence and absence of aTc.

### Construction of plasmids pUHE-YjbB, pUHE-PitA, and pUHE-Pho89

Phusion High-Fidelity DNA Polymerase (New England Biolabs) was used for the amplification of all inserts. The *pitA* and *yjbB* genes were amplified from *S. enterica* 14028s chromosome with primer pairs W3394/W3395 and W3392/W3393, respectively. PCR products were digested with BamHI and PstSI and subsequently ligated into pUHE-21 (72), previously digested with the same enzymes, to yield plasmids pUHE-PitA (pPitA) and pUHE-YjbB (pYjbB). The *pho89* was amplified from *S. cerevisiae* DY1457 (73) chromosome using primer pair W3831/W3832. PCR product was subsequently cloned into linearized pUHE-21 (72) using NEBuilder HiFi DNA Assembly Cloning Kit (New England BioLabs) to produce pUHE-Pho89 (pPho89). All constructs were verified by Sanger DNA sequencing with primers 146 and 156.

### Physiological experiments growth conditions

Physiological experiments were carried out at 37°C with shaking at 250 rpm in MOPS medium (74) supplemented with 22 mM glucose, 5 mM MgSO_4_, an amino acids mixture (1.6 mM of alanine, glycine, leucine, glutamate, and serine; 1.2 mM glutamine, isoleucine, and valine; 0.8 mM arginine, asparagine, aspartate, lysine, phenylalanine, proline, threonine, and methionine; 0.4 mM histidine and tyrosine; and 0.2 mM cysteine and tryptophan), and the indicated concentration of P source. For experiments on solid media supplemented with different P sources, 1.5% (wt/vol) noble agar (Difco) was added into MOPS minimal medium described above supplemented with 0.5 mM of the indicated P source. Bacteria were incubated for 18–24 h at 37°C. In P-shifting experiments, the cells were propagated in MOPS medium containing 1 or 2 mM K_2_HPO_4_, washed three times in MOPS medium lacking a P source, and shifted to fresh MOPS medium lacking P or supplemented with the indicated P source. The following organic phosphorus sources were used at 1 mM: adenosine 5′-triphosphate disodium salt, fructose-6-phosphate disodium salt, glucose-6-phosphate disodium salt, phosphoenol pyruvic acid monopotassium salt, D-(−)-3-phosphoglyceric acid disodium salt, *sn*-glycerol 3-phosphate bis(cyclohexylammonium) salt. Selection of plasmids was accomplished by the addition of ampicillin at 100 µg/mL (overnight growth) or 30 µg/mL (experimental condition), chloramphenicol at 20 µg/mL (overnight growth) or 10 µg/mL (experimental condition). Heterologous expression of proteins was achieved by supplementing cultures with the indicated concentrations of isopropyl β-d-1-thio-galactopyranoside (IPTG) and 0.5 µg/mL of anhydrotetracycline (aTc).

### Monitoring gene expression via fluorescence

Following inoculation into fresh MOPS medium, cultures were aliquoted as technical replicates or triplicates into black, clear-bottom, 96-well plates (Corning). Two drops of mineral oil were used to seal the wells and prevent evaporation, and cultures were grown at 37°C with auto-mixing in either a SpectraMax i3x (Molecular Devices) or a Synergy H1 (BioTek) plate reader. The green fluorescence (excitation 485 nm/emission 535 nm) and absorbance at 600 nm (OD_600_) from the wells of the plates were read at regular time intervals. Fluorescence measurements were normalized by the OD_600_ of the samples.

### Image acquisition, analysis, and manipulation

Plate images were acquired either using a blue light transilluminator/orange filter (Clare Chemical Research) and photographed using a smartphone camera or with an Azure 600 Imager (Azure). ImageJ software (75) was used to crop the edges, rotate, and adjust the brightness and contrast of the images. These modifications were simultaneously performed across the entire set of images.

### Statistical analyses

Results obtained from at least three independent experiments were plotted using GraphPad PRISM. End point data were analyzed in GraphPad PRISM using two-way ANOVA calculated with Dunnett multiple-comparison test. Kinetic data were analyzed with the modified chi-squared method as described (44).

## SUPPLEMENTAL MATERIAL

Figure S1 mbio.01642-24-s0001.tif Effect of *pitA* deletion on the activity of the PpstS-gfp transcriptional fusion.

Figure S2 mbio.01642-24-s0002.tif Growth of strains depicted in Fig. 2B.

Figure S3 mbio.01642-24-s0003.tif Effect of growth rate on the activity of the P*pstS-gfp* transcriptional fusion.

Figure S4 mbio.01642-24-s0004.tif aTc-dependent growth of *tetRA-ugpBAECQ* on plates containing or lacking specific P sources.

Figure S5 mbio.01642-24-s0005.tif Activation of PhoB/PhoR by cytoplasmic magnesium starvation and NaCl shock.

Supplemental Material mbio.01642-24-s0006.docx Supplemental tables and figure legends.

ASM does not own the copyrights to Supplemental Material that may be linked to, or accessed through, an article. The authors have granted ASM a non-exclusive, world-wide license to publish the Supplemental Material files. Please contact the corresponding author directly for reuse.
