# Supplementary figures and images for: An intracellular phosphorus-starvation signal activates the PhoB/PhoR two-component system in Salmonella enterica
Source: mBio. 2024 Aug 6;15(9):e01642-24. doi: 10.1128/mbio.01642-24 (PMC11389368; doi:10.1128/mbio.01642-24)

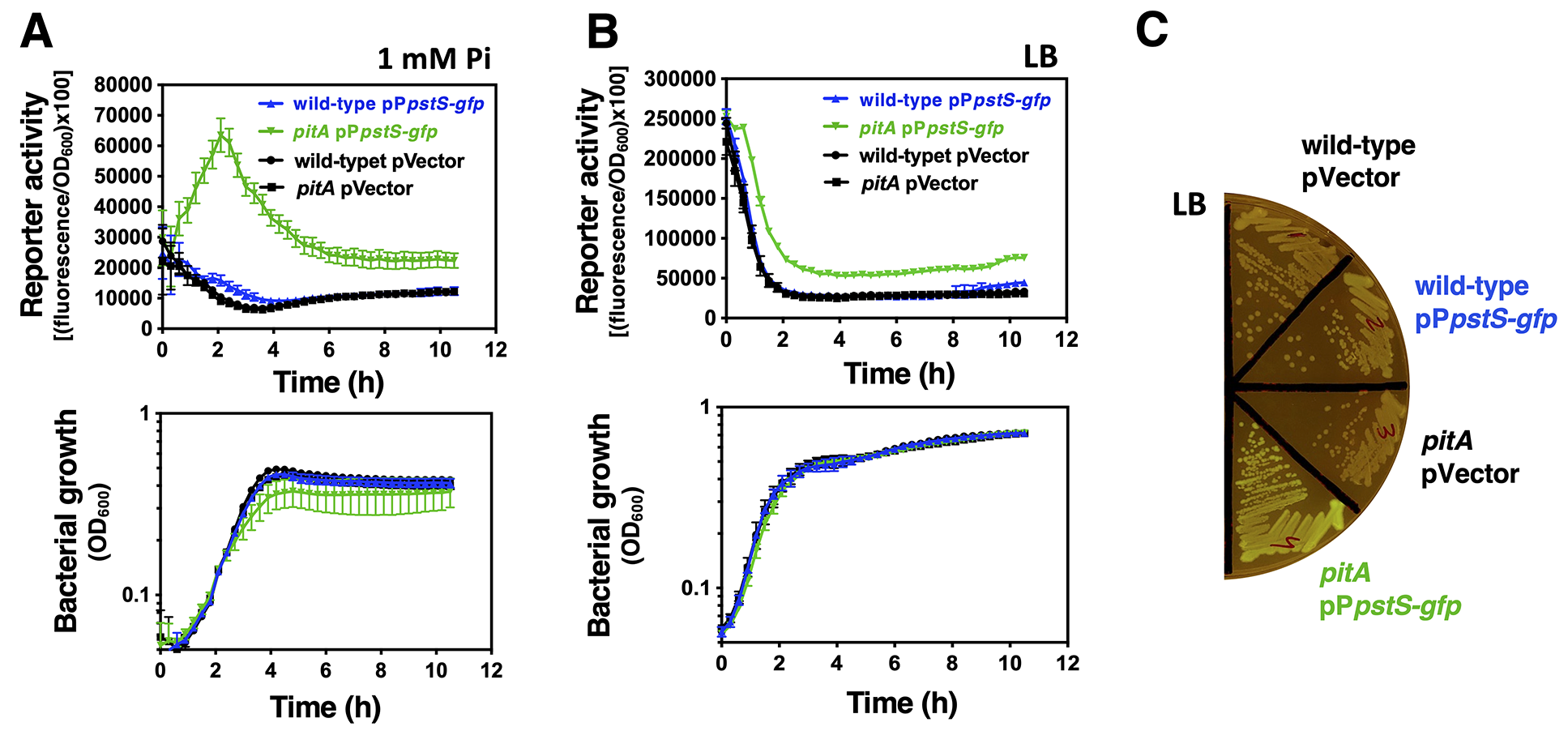

Supplement: Figure S1 — Effect of pitA deletion on the activity of the PpstS-gfp transcriptional fusion. [file mbio.01642-24-s0001.tif]

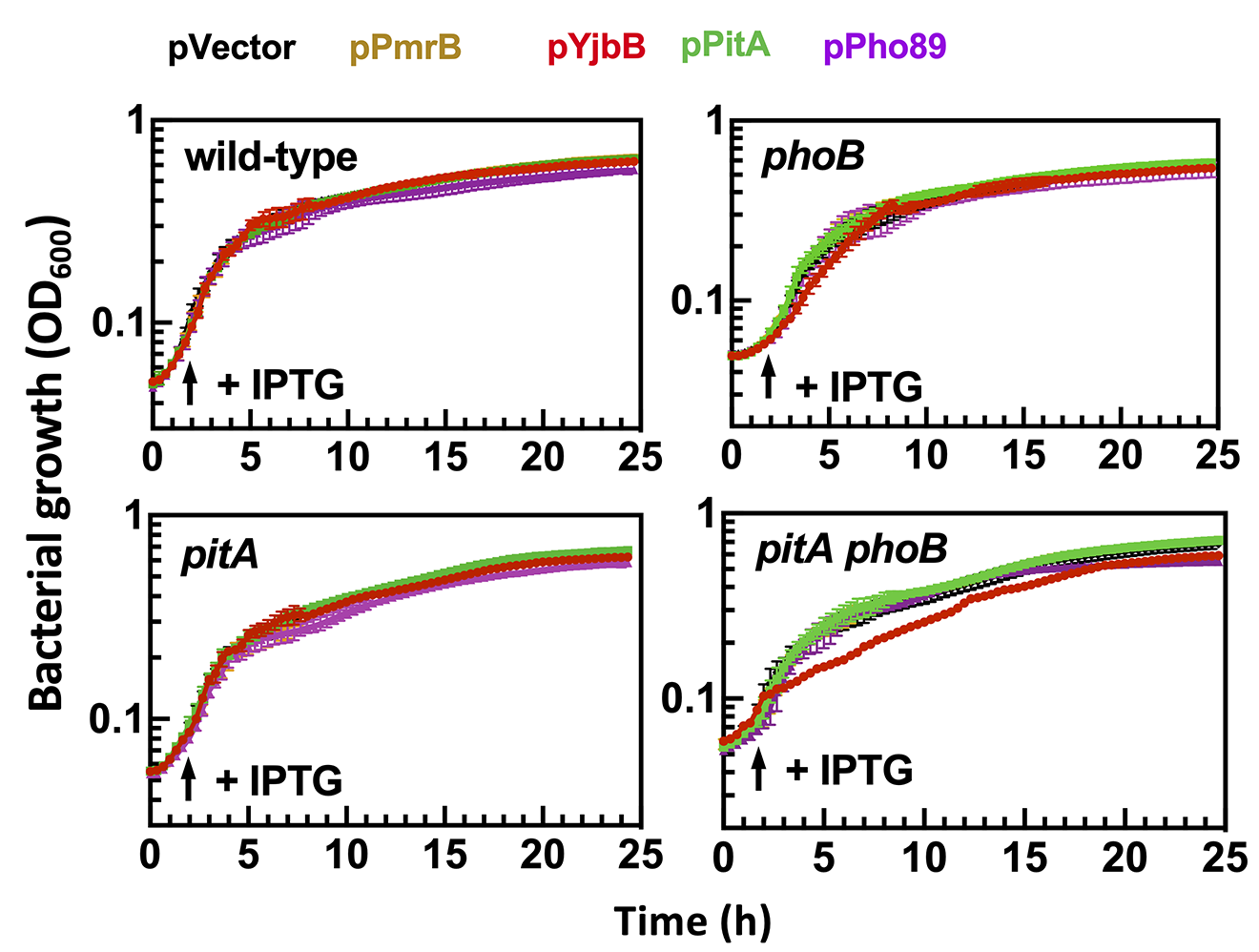

Supplement: Figure S2 — Growth of strains depicted in Fig. 2B. [file mbio.01642-24-s0002.tif]

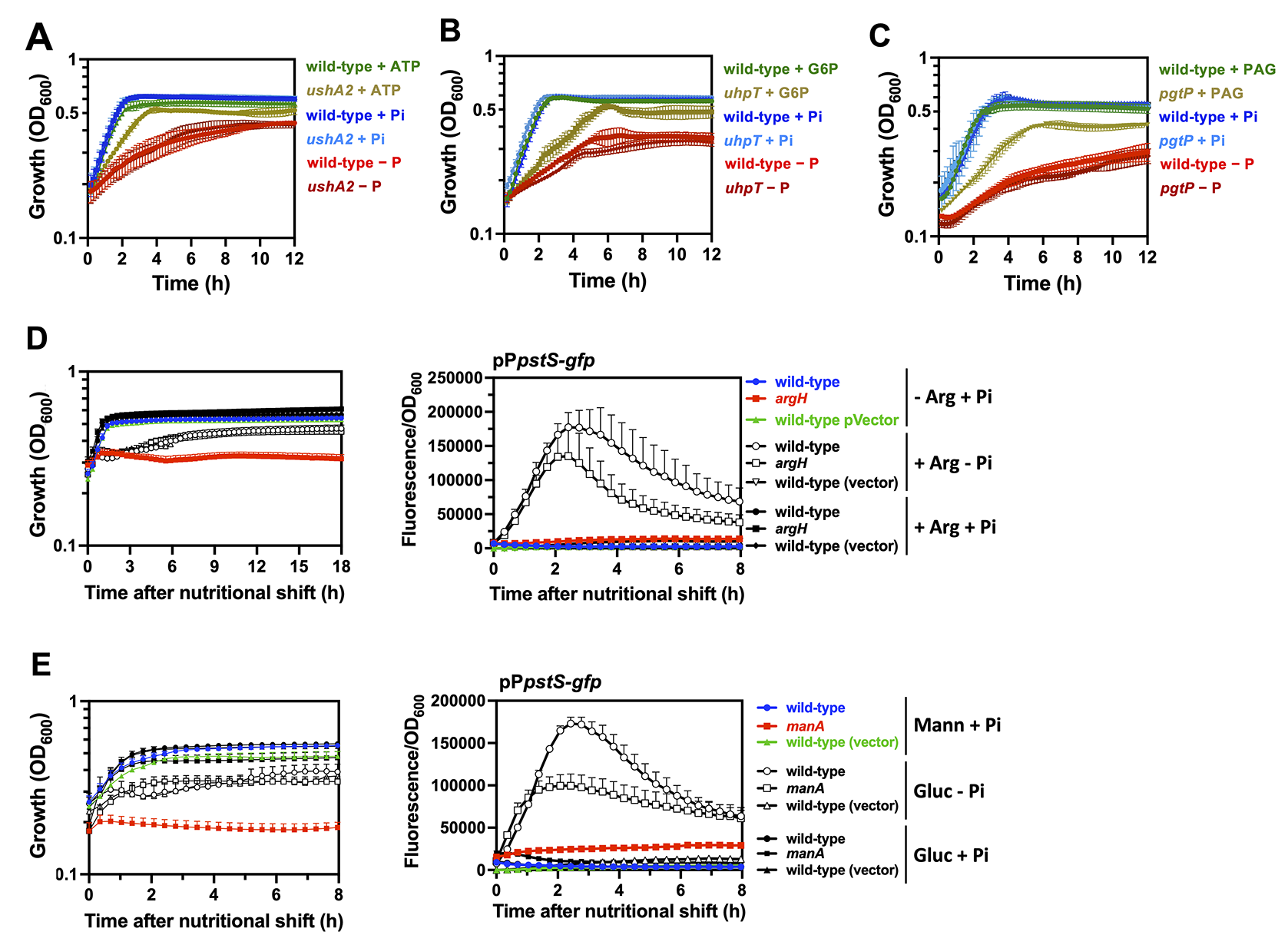

Supplement: Figure S3 — Effect of growth rate on the activity of the PpstS-gfp transcriptional fusion. [file mbio.01642-24-s0003.tif]

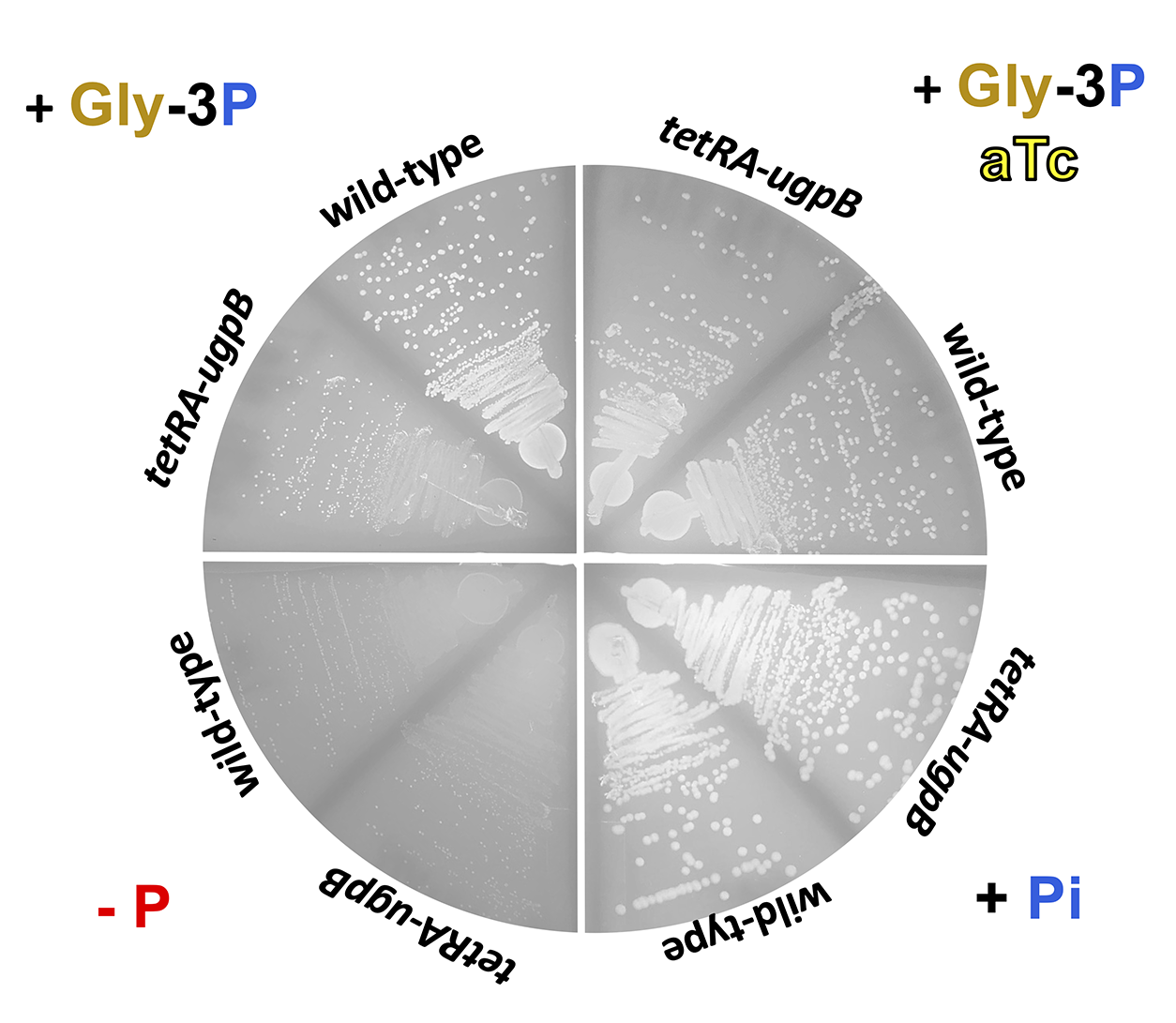

Supplement: Figure S4 — aTc-dependent growth of tetRA-ugpBAECQ on plates containing or lacking specific P sources. [file mbio.01642-24-s0004.tif]

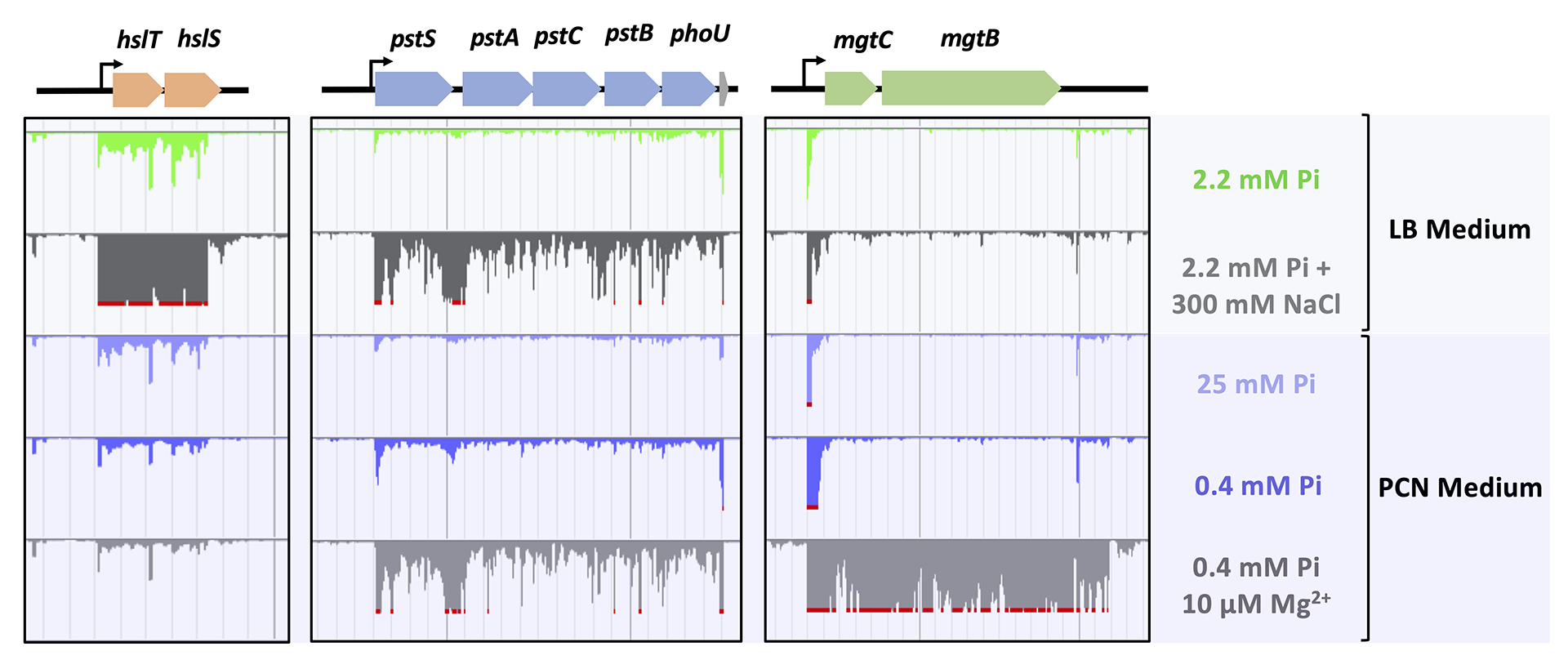

Supplement: Figure S5 — Activation of PhoB/PhoR by cytoplasmic magnesium starvation and NaCl shock. [file mbio.01642-24-s0005.tif]
