# Supplementary material for: An intracellular phosphorus-starvation signal activates the PhoB/PhoR two-component system in Salmonella enterica
Source: mBio. 2024 Aug 6;15(9):e01642-24. doi: 10.1128/mbio.01642-24 (PMC11389368; doi:10.1128/mbio.01642-24)
Supplement: Supplemental Material — Supplemental tables and figure legends. [file mbio.01642-24-s0006.docx]

**Supplemental Material**

**Table S1. Bacterial strains and plasmids used in this study.**

| **Strain** | **Relevant characteristics** | **Identifier** | | **Source** | |  |
| --- | --- | --- | --- | --- | --- | --- |
| ***Escherichia coli*** | | | | | | |
| EC100D | *pir*^+^ (DHFR) host strain used for generation and propagation of plasmid constructs | N/A | Epicentre | |  |  |
|  |  |  |  | |  |  |
| ***Salmonella enterica* serovar Typhimurium** | | | | | | |
| 14028s | wild-type | wild-type | | [1] | |  |
| EG9054 | *phoB*::Km^R^ | *phoB* | | [2] | |  |
| MP2256 | *ΔphoR*::Km^R^ | *phoR* | | This study | |  |
| MP1251 | *ΔpitA::*Ap^R^ | *pitA* | | [3] | |  |
| MP1252 | *ΔyjbB::*Km^R^ | *yjbB* | | [3] | |  |
| MP2405 | *phoB*::Km^R^ *ΔpitA::*Ap^R^ | *phoB pitA* | | This study | |  |
| EG6537 | *argH::*Tn*10* | *argH* | | Lab strain | |  |
| MP50 | *ΔmanA::*Cm^R^ | *manA* | | [4] | |  |
| MP2133 | Km^R^*-tetRA-ugpBAECQ* | *teRA-ugpBAECQ* | | This study | |  |
| MP2285 | Km^R^*-tetRA-ugpBAECQ ΔyjbB ΔglpT::*Gm^R^ *ΔpitA::*Ap^R^ | *teRA-ugpBAECQ yjbB glpT pitA* | | This study | |  |
| MP1737 | *ΔglpT::*Gm^R^ | *glpT* | | [5] | |  |
| MP1738 | *ΔuhpT::*Ap^R^ | *uhpT* | | [5] | |  |
| MP1739 | *ΔpgtP::*Tn*10* (Tet^R^) | *pgtP* | | [5] | |  |
| MP1779 | *ΔushA2*::Gm^R^ | *ushA2* | | [5] | |  |
| MP2262 | *ΔpgtP::*Tn*10* *phoB*::Km^R^ | *pgtP phoB* | | This study | |  |
| MP2263 | *ΔpgtP::*Tn*10* *ΔphoR*::Km^R^ | *pgtP phoR* | | This study | |  |
| MP2264 | *ΔuhpT::*Ap^R^  *phoB*::Km^R^ | *uhpT phoB* | | This study | |  |
| MP2265 | *ΔuhpT::*Ap^R^  *ΔphoR*::Km^R^ | *uhpT phoR* | | This study | |  |
| MP2260 | *ΔushA2*::Gm^R^ *phoB*::Km^R^ | *ushA2 phoB* | | This study | |  |
| MP2261 | *ΔushA2*::Gm^R^ *ΔphoR*::Km^R^ | *ushA2 phoR* | | This study | |  |
|  |  |  | |  | |  |
| **Plasmids** |  |  | |  | |  |
| pSIM6 | rep_pSC101_^ts^ Amp^R^ P_CI857_-γβexo | pSIM6 | | [6] | |  |
| pKD4 | rep_R6Kγ_ Amp^R^ FRT Km^R^ FRT | pKD4 | | [7] | |  |
| pKD4-Ap^R^ | rep_R6Kγ_ Amp^R^ FRT Ap^R^ FRT | pKD4-Ap^R^ | | [4] | |  |
| pKD4-Gm^R^ | rep_R6Kγ_ Amp^R^ FRT Gm^R^ FRT | pKD4-Gm^R^ | | [4] | |  |
| pKD4-Tn*10* | rep_R6Kγ_ Amp^R^ FRT Tn*10* (Tet^R^) FRT | pKD4-Tn*10* | | [4] | |  |
| pCP20 | rep_pSC101_^ts^ λ cI857 FLP Amp^R^ Cm^R^ | pCP20 | | [7] | |  |
| pBbB2K-GFP | rep_pBBR1_ Km^R^ *tetRA-gfp* | pBbB2K-GFP | | [8] | |  |
| pGFP | rep_p15A_ Cm^R^ promoterless *gfp* vector control | pACYC-GFPc, pVector | | [2] | |  |
| pP*pstS*-*gfp* | rep_p15A_ Cm^R^ P*pstS*-*gfp* | pACYC-P*pstS*-GFPc | | [2] | |  |
| pFPV25 | rep_pMB1_ Amp^R^ promoterless *gfp* vector control | pFPV25, pVector | | [9] | |  |
| pP*pstS*-*gfp* | rep_pMB1_ Amp^R^ P*pstS*-*gfp* | pFPV-PstS | | [2] | |  |
| pUHE-21–2-*lacI^q^* | rep_pMB1_ lacI^q^ Amp^R^; vector control | pUHE-21, pVector | | [10] | |  |
| pUHE-YjbB | rep_pMB1_ lacI^q^ Amp^R^ P*lac-yjbB* | pYjbB | | This work | |  |
| pUHE-PitA | rep_pMB1_ lacI^q^ Amp^R^ P*lac-pitA* | pPitA | | This work | |  |
| pUHE-Pho89 | rep_pMB1_ lacI^q^ Amp^R^ P*lac-pho89* | pPho89 | | This work | |  |
| pUHE-PmrB | rep_pMB1_ lacI^q^ Amp^R^ P*lac-pmrB* | pPmrB | | [11] | |  |

Amp^R^ – ampicillin resistance; Ap^R^ – apramycin resistance; Cm^R^ – chloramphenicol resistance; Gm^R^ – gentamycin resistance; Km^R^ – kanamycin resistance.

**Table S2. Oligonucleotides sequences used in this study**

| **Name** | **Sequence (5’→ 3’)** | **Purpose** |
| --- | --- | --- |
| 143 | TGTCCAGATAGCCCAGTAGC | Verification of *ΔphoR*::*km^R^* |
| 1173 | TACAGTCGCGAGCAGCTACT | Verification of *ΔphoR*::*km^R^* |
| 2075 | GGCGGCTTACGCTGGAGCAAATTGCCGTTCGCTTACGTGCCTGGGCACTAGTGCTTGGAT | Generation of *km^R^-tetRA- ugpBAECQ* |
| 2076 | ACTCAGCGCCAGTCCTAAAGCTGTATGTCGTAACGATATCATATGTATATCTCCTTCTTA | Generation of *km^R^-tetRA- ugpBAECQ* |
| 2077 | CCTTCGATTCCGACCTCA | *km^R^-tetRA- ugpBAECQ* verification |
| 2078 | GCGCTCAGGTTCTGTTCA | *km^R^-tetRA- ugpBAECQ* verification |
| 2079 | CGCATCGCCTTCTATCGC | *km^R^-tetRA- ugpBAECQ* verification |
| 2080 | ACTCGCTGCGAATCCGGAT | *km^R^-tetRA- ugpBAECQ* verification |
| W3392 | TATCGCAGGATCCACGTATAACGATAAGGAGAAC | *yjbB* cloning into pUHE-21–2-*lacI*^q^ |
| W3393 | TATCGCACTGCAGATGACGGGCCTACGCGCATG | *yjbB* cloning into pUHE-21–2-*lacI*^q^ |
| W3394 | TATCGCAGGATCCGATAATGCGCCGCGTTCATG | *pitA* cloning into pUHE-21–2-*lacI*^q^ |
| W3395 | TATCGCACTGCAGTTAAATAATCTTCAGGGAA | *pitA* cloning into pUHE-21–2-*lacI*^q^ |
| W3831 | TTAGCCCTCTGGCAAATCGTTTTCTAAGGACTTTAAGAAGGAGATATACATATGGCTTTACATCAATTTGA | *pho89* cloning into pUHE-21–2-*lacI*^q^ |
| W3832 | GATCTATCAACAGGAGTCCAAGCTCAGCTAATTAAGTTATGTCATTTGGTATTCC | *pho89* cloning into pUHE-21–2-*lacI*^q^ |
| 146 | AATCCAGATGGAGTTCTGAGG | pUHE-21–2-*lacI*^q^ sequencing |
| 153 | GTCTCATGAGCGGATACATAT | pUHE-21–2-*lacI*^q^ sequencing |
|  |  |  |
|  |  |  |
|  |  |  |
|  |  |  |
|  |  |  |
|  |  |  |

**Supplemental Figure Legends**

**Figure S1. Effect of *pitA* deletion on the activity of the P*pstS-gfp* transcriptional fusion. (A)** (Top) Fluorescence and (bottom) growth of wild-type (14028s) and *pitA* (MP1251) strains of *Salmonella* harboring pP*pstS-gfp* (pFPV-PstS) or the vector control (pVector, pFPV25). Measurements were performed during growth in MOPS glucose medium supplemented with 1 mM Pi (K_2_HPO_4_). **(B)** (Top) Fluorescence and (bottom) growth of strains described in (A). Measurements were performed in LB medium, which contains 2.2 mM Pi [12]. **(C)** Fluorescence of strains described in (A) on LB plates. Plates were incubated at 30°C for 14-16 h. Means ± SDs of at least three independent experiments are shown. Image is representative of three independent experiments.

**Figure S2. Growth of strains depicted in Fig. 2B.** Wild-type (14028s), *phoB* (EG9054), *pitA* (MP1251), and *pitA phoB* (MP2405) *Salmonella* carrying pACYC-P*pstS*-GFPc (pP*pstS*-*gfp*) and either pUHE-21 (pVector), pUHE-PmrB (pPmrB), pUHE-YjbB (pYjbB), pUHE-PitA (pPitA) or pUHE-Pho89 (pPho89). Experiments were conducted in LB broth. Plasmid expression of YjbB, PitA and Pho89 were induced after 2 h of growth by the addition of 250 μM of isopropyl β- d-1-thiogalactopyranoside (IPTG).

**Figure S3. Effect of growth rate on the activity of the P*pstS-gfp* transcriptional fusion. (A-C)** Growth from wild-type (14028s), *ushA2* (MP1779) *uhpT* (MP1738), and *pgtP* (MP1739) *Salmonella* carrying pP*pstS-gfp* under the conditions outlined in Fig. 3: Prior to performing the readings, cultures were grown to mid-logarithmic phase in MOPS medium containing 1 mM Pi (K_2_HPO_4_), washed in MOPS medium lacking a P source and resuspended in MOPS medium containing 1 mM of the indicated organic P source (ATP: adenosine triphosphate; G6-P: glucose 6-P; PGA: 3-phosphoglyceric acid), 1 mM Pi (K_2_HPO_4_) or lacking P. Means ± SDs of at least three independent experiments are shown. **(D)** (Left) growth curve and (right) corresponding fluorescence of wild-type (14028s) and *argH* (EG6537) *Salmonella* harboring pP*pstS-gfp* (pFPV-PstS) or the vector control (pVector, pFPV25). Cultures were grown to mid-logarithmic phase in MOPS medium containing 1.6 mM arginine and 1 mM Pi (K_2_HPO_4_), washed in MOPS medium lacking arginine and a P source. Cells were subsequently resuspended in either MOPS medium containing or lacking 1.6 mM arginine (+Arg; -Arg) and 1 mM K_2_HPO_4_ (+Pi; -Pi). Growth and green fluorescence were then monitored for 8 h. **(E)** (Left) growth curve and (right) corresponding fluorescence of wild-type (14028s) and *manA* (MP50) *Salmonella* harboring pP*pstS-gfp* or the vector control (pVector, pFPV25). Cultures were grown to mid-logarithmic phase in MOPS medium containing 22 mM glucose and 1 mM K_2_HPO_4_. Cells were washed in MOPS medium lacking carbon and P and then resuspended in MOPS medium containing either 22 mM mannose (+Man) or 22 mM glucose (+Glc) as C sources, and either with or without 1 mM K_2_HPO_4_ (+Pi; -Pi). Growth and green fluorescence were monitored for the next 8 h. Graphs depicted in F-G show the means ± SDs of at least three biological replicates are shown and are representatives of at least 3 experiments.

**Figure S4. aTc-dependent growth of *tetRA-ugpBAECQ* on plates containing or lacking specific P sources.** Growth of wild-type (14028s) and *tetRA-ugpBAECQ* (MP2133) *Salmonella* strains on MOPS-glucose-noble agar plates either lacking P (-P) or containing 1 mM of Gly-3P (+ Gly-3P), 1 mM of Gly-3P and 0.5 μg/ml of aTc (+ Gly-3P aTc), or 1 mM K_2_HPO_4_ (+ Pi). Plates were incubated at 37°C for 16-18 h before being imaged. A representative plate of at least three experiments is shown.

**Figure S5. Activation of PhoB/PhoR by cytoplasmic magnesium starvation and NaCl shock.** Top: Genomic regions encoding sodium chloride-responsive (*hslTS*), phosphate starvation-responsive (*pstSCAB-phoU*), and cytoplasmic magnesium starvation-responsive (*mgtCB*) genes from *Salmonella enterica*. Bottom: Graphic representation of RNA-seq reads following bacterial growth under the conditions described on the right-hand-side of the graph. Green reads are derived from bacteria grown to mid-exponential phase (OD_600_ = 0.3) in LB medium. Dark gray reads are derived from bacteria grown to mid-exponential phase (OD_600_ = 0.3) in LB medium and shocked for 10 minutes with 300 mM NaCl. LB contains 2.2 mM Pi [12]. Light blue reads are derived from bacteria grown to mid-exponential phase (OD_600_ = 0.3) in defined PCN medium containing 25 mM K_2_HPO_4_and pH of 7.4. Light blue reads are derived from bacteria grown to mid-exponential phase (OD_600_ = 0.3) in defined PCN medium containing 0.4 mM K_2_HPO_4_, 1 mM MgSO_4_ and pH of 5.8. Light gray reads are derived from bacteria grown to mid-exponential phase (OD_600_ = 0.3) in defined PCN medium containing 0.4 mM Pi, 0.01 mM MgSO_4_ and pH of 5.8. Detailed experimental conditions and original data obtain from [13].

**Supplemental Material References**

1. Fields, P.I., et al., *Mutants of Salmonella typhimurium that cannot survive within the macrophage are avirulent.* Proc Natl Acad Sci U S A, 1986. **83**(14): p. 5189-93.

2. Pontes, M.H. and E.A. Groisman, *Protein synthesis controls phosphate homeostasis.* Genes Dev, 2018. **32**(1): p. 79-92.

3. Bruna, R.E., et al., *Limitation of phosphate assimilation maintains cytoplasmic magnesium homeostasis.* Proc Natl Acad Sci U S A, 2021. **118**(11).

4. Pontes, M.H. and E.A. Groisman, *Slow growth determines nonheritable antibiotic resistance in Salmonella enterica.* Sci Signal, 2019. **12**(592).

5. Bruna, R.E., C.G. Kendra, and M.H. Pontes, *Phosphorus starvation response and PhoB-independent utilization of organic phosphate sources by Salmonella enterica.* Microbiol Spectr, 2023: p. e0226023.

6. Datta, S., N. Costantino, and D.L. Court, *A set of recombineering plasmids for gram-negative bacteria.* Gene, 2006. **379**: p. 109-15.

7. Datsenko, K.A. and B.L. Wanner, *One-step inactivation of chromosomal genes in Escherichia coli K-12 using PCR products.* Proc Natl Acad Sci U S A, 2000. **97**(12): p. 6640-5.

8. Lee, T.S., et al., *BglBrick vectors and datasheets: A synthetic biology platform for gene expression.* J Biol Eng, 2011. **5**: p. 12.

9. Valdivia, R.H. and S. Falkow, *Bacterial genetics by flow cytometry: rapid isolation of Salmonella typhimurium acid-inducible promoters by differential fluorescence induction.* Mol Microbiol, 1996. **22**(2): p. 367-78.

10. Soncini, F.C., E.G. Vescovi, and E.A. Groisman, *Transcriptional autoregulation of the Salmonella typhimurium phoPQ operon.* J Bacteriol, 1995. **177**(15): p. 4364-71.

11. Wosten, M.M., et al., *A signal transduction system that responds to extracellular iron.* Cell, 2000. **103**(1): p. 113-25.

12. Spira, B., et al., *Alternative promoters in the pst operon of Escherichia coli.* Mol Genet Genomics, 2010. **284**(6): p. 489-98.

13. Kroger, C., et al., *An infection-relevant transcriptomic compendium for Salmonella enterica Serovar Typhimurium.* Cell Host Microbe, 2013. **14**(6): p. 683-95.
